# Oriental pied hornbills (*Anthracoceros albirostris*) solve invisible displacement tasks in a test of Piagetian object permanence

**DOI:** 10.1098/rsbl.2023.0547

**Published:** 2024-01-31

**Authors:** Ruitong Yao, Elias Garcia-Pelegrin

**Affiliations:** Department of Psychology, National University of Singapore, Singapore 117572, Singapore

**Keywords:** object permanence, Bucerotiformes, avian cognition, Oriental pied hornbill

## Abstract

Object permanence, the ability to mentally represent objects even when they are not directly accessible to the senses, is of vital importance for the survival of both human and non-human animals. The Oriental pied hornbill (*Anthracoceros albirostris*) is an Asian species of hornbill displaying remarkable adaptability in various environments, yet little is known about their cognitive abilities. Their breeding behaviour is unique, as the female hornbill seals herself inside a cavity before laying eggs and the male feeds her and their offspring without visual contact, strongly suggesting the presence of object permanence to some degree. In this study, six Oriental pied hornbills underwent testing for object permanence, including a series of seven standard Piagetian tasks involving visible and invisible displacements. The subjects consistently demonstrated spontaneous object permanence in all stages leading up to the invisible displacement stage. Half of the subjects achieved full stage 6 double invisible displacement Piagetian object permanence, while the other half reached stage 5 double visible displacement. Breeding behaviour and the duration of developmental stages are proposed as potential factors influencing object permanence ability in this species of hornbill.

## 1. Introduction

We perceive objects as entities that persist through time and space. If our view of an object is obstructed, we know of its existence though we cannot see it. This is because we understand that an object still exists even when it is not accessible to our senses. Object permanence [1] is an essential developmental milestone in cognition because it is closely linked to the understanding of the concepts of space, time and causality [1–3]. Without object permanence, objects would keep disappearing and reappearing unexpectedly; we would attribute everything we experienced to our own actions; and the boundary between ourselves and the external world would be blurred. From an evolutionary perspective, the ability to represent other animals and objects when they are out of sight provides great adaptive advantages in activities such as foraging and avoiding predation. For example, prey hiding from the predator in a tree crevice will have a greater chance of surviving if the prey knows that the predator still exists outside of the crevice even though it is currently out of sight [4–6].

Piaget [1] defined six stages of object permanence (table 1 for an explanation of these stages), which are progressive in difficulty, that human infants would experience during the development in their first 2 years. Visible and invisible displacement are the two main types of tasks used to test for object permanence. Outside of human understanding of object permanence, the Piagetian framework has been widely adapted in comparative cognition to study how non-human animals fare under similar object permanence tasks, and how such understanding develops among different species (e.g. [7–11]). The comparison between species can reveal how the same cognitive ability has evolved in unrelated species and what species-specific characteristics have affected its development [12–14]. The similarities among species may contribute to our understanding of the cognitive ability of their common ancestor, while the differences may highlight selection pressures necessary for the development of cognitive ability [15].

**Table 1.** Experimental tests.

test	description	
stage 1	Subjects do not respond to the object.	
stage 2	Subjects visually track the movement of the object.	
stage 3	In this test, the experimenter showed the subject a reward which was partially hidden under one of the cups, so that half of the reward was still visible to the subject.	
stage 4	This test was similar to stage 3, but now the reward was fully hidden under one of the cups.	
A not B test	This test was designed to assess ‘A-not-B errors.’ Initially, a reward was shown to the subject and completely hidden under one of the three cups (cup A). The hornbill was then allowed to peck at only one prong. If the hornbill picked the correct location (cup A) in two consecutive trials, the reward would then be hidden under a second cup (cup B). Successful completion of this test involved the bird selecting the prong corresponding to the cup where the reward was ultimately concealed (cup B) during the initial presentation. The bird searching under the first cup (cup A) would indicate an ‘A-not-B error.’	
stage 5a	The reward was initially placed under one cup (as in stage 4), then visibly moved to another cup.	single visible displacement
stage 5b	This test was similar to the single visible displacement but with an additional cup involved in moving the reward. After the reward was visibly moved to second cup, it was visibly moved to a third cup.	double visible displacement
stage 6a	The reward was initially concealed beneath a smaller cup, which was then visibly displaced to one of the main locations and covered with the bigger experimental cup. At this juncture, the experimenter emptied the contents of the smaller cup into the experimental cup with one hand, while simultaneously obscuring the manoeuvre with the other. Finally, the experimenter revealed the now-empty interior of the smaller cup to the subject (see electronic supplementary material, video).	single invisible displacement
stage 6b	Similar to single invisible displacement but with the additional step of moving the smaller cup inside a second cup after the first move. The insides of the smaller cup would be emptied inside the second cup (see electronic supplementary material, video).	double invisible displacement

Note. Stages 1 and 2 were not tested in the current study.

Previous research on non-human primates suggests that most primates can solve visible displacement tasks, but only the great apes have been reported to solve invisible displacement tasks, the highest level of object permanence, with compelling evidence [7,8,16–23]. In avian species, most of the parrots and corvids tested have been able to pass invisible displacement tasks (e.g. [24–26]). However, outside of the Passeriformes and Psittaciforme order, no other avian species has been reported to reach such a level of understanding, with both ring doves (*Streptopelia risoria*) and tits (*Periparus ater* and *Parus major*) reaching stage 4, and some birds like horned puffins (*Fratercula corniculata*) having only reached stage 3 [27–29]. The observation that both parrots and corvids exhibit a superior level of object permanence compared to other bird species aligns with the prevailing consensus that these avian groups have evolved advanced cognitive abilities. These include delayed gratification, inference-making, and tool usage, which require high levels of object permanence, surpass the cognitive capacities of most other bird species studied so far, and seem comparable to the cognitive abilities of non-human apes [30–32]. For example, apes, parrots, and corvids can refrain from an immediate reward in exchange for a better but delayed reward [33–35]. They have also demonstrated inference by exclusion in the visual domain in object-choice tasks [36–38]. Corvids have demonstrated episodic-like memory capability by remembering the *what*, *where*, and *when* qualities of an event [39]. Moreover, like apes, some cases of parrots and corvids manufacturing and using tools to retrieve food or water have been reported (e.g. [40–42]). Though parrots and corvids have very different brain structures than apes, they have developed similar cognitive abilities as a result of convergent evolution, likely because they must overcome analogous challenges in their respective environments [43]. Indeed Emery [43] concluded that the emergence of sophisticated cognitive abilities is associated with specific prerequisites including an omnivorous diet, social behaviour, large relative brain size, innovation, extended development, longevity, and habitat adaptability. Parrot and corvid species seem to have fulfiled most of these preconditions. For instance, the relative brain size of parrots and corvids is larger than most other birds and comparable to primates [44]. They have also demonstrated high frequencies of innovations in foraging by eating novel food or obtaining food with new strategies [45]. These preconditions have driven them to adapt to variable environments, and such adaptability is associated with the evolution of intelligence [30,43,46].

Similar to parrots and corvids, hornbills (family Bucerotidae, order Bucerotiformes) fulfil many preconditions described by Emery [43]. Primarily distributed in sub-Saharan Africa and southern Asia, hornbills exhibit an extended developmental period, encompassing 25–40 days of incubation and 45–86 days as nestlings. In captivity, smaller hornbill species typically enjoy a lifespan of over 20 years, while larger species can live for up to 50 years [47]. Notably, their relative brain size surpasses that of some parrot species [44]. Many hornbill species display high levels of sociality, frequently engaging in social play, a behaviour associated with enhanced cognitive abilities [48–50]. To this day, our understanding of the cognitive capabilities of hornbills, with a specific focus on Asian hornbills, remains limited. Among the Asian hornbills, Oriental pied hornbills (*Anthracoceros albirostris*) have demonstrated incredible adaptability. All Asian hornbill species typically only inhabit forests; however, Oriental pied hornbills have been able to adapt to both semi-urban and rural areas [51,52], with reports of some individuals even nesting in clay jars instead of their usual tree crevice [53]. They have also adapted to a more diverse diet. While most hornbill species are frugivorous, Oriental pied hornbills are omnivorous and often also predate on nests of other birds [43,54,55]. Their ability to adapt to variable environments has enabled them to overcome challenges, such as habitat loss and degradation, and to thrive in the urban environment in Singapore [51,55]. Alongside this, Asian hornbills such as the Oriental pied hornbill are often known for their unusual breeding behaviour. Most of them are monogamous. The female will enter a tree cavity and seal the entrance with a mixture of droppings, mud, food remains and saliva, leaving only a narrow opening. The male is responsible for bringing food to the female and their offspring through the opening for a few months until the offspring fledge [47,56]. The male identifies the cavity either by vocalization or the fresh seeds from fruits consumed and droppings near the tree [56]. The female may leave the nest to assist the male in feeding the young, with fledglings subsequently resealing the nest cavity after the female's departure [47]. There may be intruding conspecifics attempting to take over the nest, and the male will need to defend it, mainly by sealing the nest with more mud. This unique breeding behaviour may have evolved due to the need to protect the nest from both conspecific and heterospecific invaders [57]. Drawing from this comportment, it seems that hornbills could gain an advantage from a certain degree of understanding that the female and offspring are situated within the cavity, even in the absence of direct visual confirmation. This conduct hints at the potential existence of object permanence in this particular bird species. Consequently, the present study aims to expand the current understanding of object permanence in the avian taxa, by investigating how a seldom studied species of bird, the Oriental pied hornbill, performs in a battery of object permanence tasks.

## 2. Methods

### (a) Subjects and housing

Six Oriental pied hornbills (*Anthracoceros albirostris*) (three females) participated in this study. The subjects belong to the Mandai Wildlife Reserve Collection and are housed either in pairs or individually for unpaired subjects in aviaries measuring (approximately 6 × 4 × 3 m). The bird subjects in the study were either rescued from illegal trade or housed at the zoo because they were unable to survive in the wild, likely due to permanent physical injuries sustained before fledging. Although their exact ages are unknown, it is estimated that all subjects fell within the 5- to 10-year-old range. The hornbills were fed a maintenance diet of fresh fruit and always had ad libitum access to water.

### (b) Procedures

The experiments were reviewed and approved by the National University of Singapore's Institutional Animal Care and Use Committee (IACUC protocol number R23-0737) and The Mandai Wildlife Reserve research panel.

### (c) Pre-training

**Figure 1.**
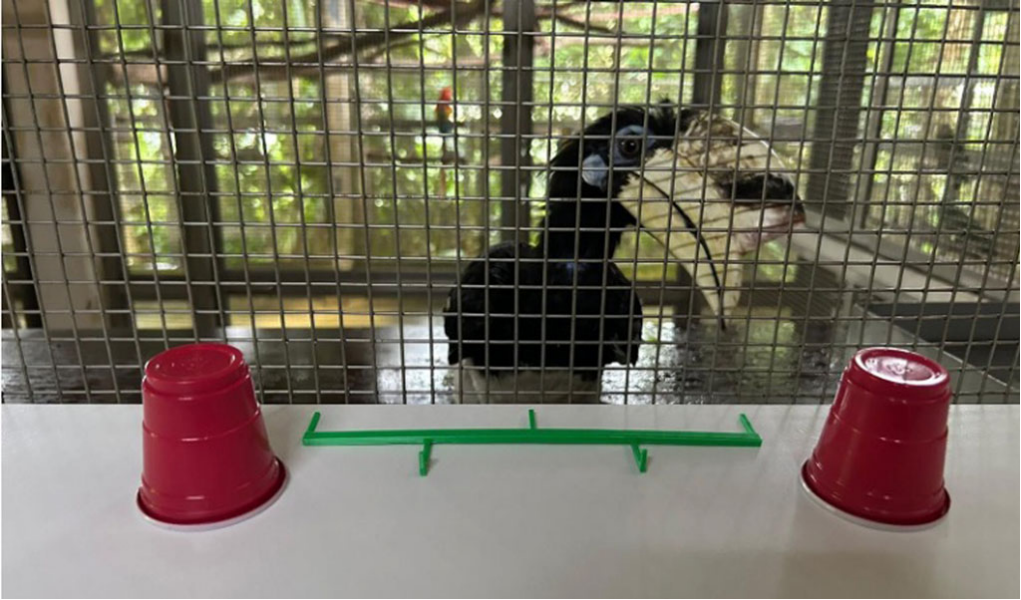
Picture of the experimental set-up. This image is a demonstration of the types of cups and choicer used. Three cups were used in the actual experiment.

The hornbill subjects were previously trained to use a tool called the ‘Choicer’ (a 6-inch polylactic acid (PLA) bar with three prongs that can pass through the enclosure mesh) to indicate where they considered food was located among three possible locations (figure 1). Before the experiment, the subjects were already proficient in using the Choicer to find fully visible rewards, systematically scoring 12/12 correct trials in training batteries. However, they had not encountered situations where the reward was partially or fully hidden under an object. Before each testing session, the hornbills received a refresher involving five trials with visible rewards. If a hornbill failed more than one trial during the refresher, testing was postponed until the next day.

### (d) Object permanence testing

The experiment consisted of seven Piagetian object permanence tests ranging from stages 3 to 6 (table 1 and electronic supplementary material, movie S1). Each test (except for the A not B test) had a minimum of one session with 12 trials. To progress to the next test, the hornbill needed to complete at least 10 correct trials in a single session. If this criterion was not met after 10 sessions, testing with that bird ended. The A-not-B test was conducted after the hornbill passed stage 4, as part of the progression from stages 3 to 6. The number of trials per session was not fixed but dependent on the performance of the hornbill. The passing criterion of the A-not-B test was three consecutive correct sessions within 10 sessions. For each test trial, the experimenter directed the hornbill to position itself at the Choicer's centre with the food reward, with the prongs outside its enclosure. The Choicer had cups behind its prongs, hiding rewards. The hornbill was allowed to watch the apparatus being set up. To mitigate the potential influence of hand tracking confounds, incorrect cups were frequently touched, before and after the display. After demonstrating, the Choicer was moved forward, making the prongs accessible. The hornbill was allowed to peck at only one prong, and success was based on choosing the prong with the reward. The reward was given to the hornbill when its bill touched the correct prong. No reward was given if it pecked at the wrong prong. During the trial, if the hornbill moved away from the set-up or lost attention, the experimenter would direct the hornbill back to the position with the food reward and restart the trial. The reward was either a food pellet or a small piece of fruit and its location was pseudo-randomized in each trial.

### (e) Analysis

All analysis was conducted using RStudio [58]. We analysed subject performance during the initial sessions of each stage, which serves as an indicator of their spontaneous object permanence. For each stage, binomial tests and Wilcoxon's tests were used to evaluate the performance in comparison to expectation (0.33) on both the individual level and group level respectively. A generalized linear mixed model (GLMM) and *post-hoc* Tukey HSD tests were used to assess the effects of stages, trials, breeding experience (whether the subject has ever mated before, in the wild or in the captivity) and sex on performance of all sessions, with subject as a random effect (lme4 package [59] and emmeans package [60]).

## 3. Results and discussion

Among the six Oriental pied hornbills tested, three achieved full stage 6 double invisible displacement Piagetian object permanence, and the remaining three achieved stage 5 double visible displacement. To the best of our knowledge, Oriental pied hornbills are the first bird species outside of the corvid and parrot families to display object permanence levels comparable to apes.

The subjects’ performance remained consistent across trials (GLMM, *p* = 0.88), indicating that their proficiency was not due to learning or practice during the study, but rather a genuine comprehension of object permanence. Among the subjects that completed a stage, they completed it in an average of 1–3 sessions (table 2), and they also exhibited spontaneous object permanence in all stages leading up to the invisible displacement stage (Wilcoxon's tests, *p* < 0.05; table 2). All subjects passed the A-not-B task within an average of 4.5 trials. The maximum number of trials required was 6. The presence of advanced object permanence capabilities in Oriental pied hornbills is not surprising, as it likely plays a crucial role in the breeding behaviour of both males and females. This could explain the evolutionary selection for a high degree of object representation and the absence of significant differences in performance between male and female subjects (GLMM, *p* = 0.78).

**Table 2.** Test results.

A) number of sessions required to reach the criterion of stage 3–6b						
subjects (sex)	stage					
	3	4	5a	5b	6a	6b
Chika (M)	2	1	3	1	1	1
Chiku (F)	1	2	5	2	5	1
Olivia (F)	2	2	1	5	N/A	N/A
Oscar (M)	4	1	1	4	N/A	N/A
Sam (M)	1	1	2	1	N/A	N/A
Yaz (F)	1	3	6	3	3	1
average	1.83	1.67	3	2.67	3	1
B) number of correct responses in the first session for stage 3–6b						
	stage					
subjects	3	4	5a	5b	6a	6b
Chika	9*	11*	9*	11*	10*	11*
Chiku	11*	9*	8*	9*	6	11*
Olivia	8*	9*	10*	8*	4	N/A
Oscar	9*	11*	10*	7	4	N/A
Sam	12*	11*	9*	12*	3	N/A
Yaz	12*	8*	6	9*	5	12*
*p* (Wilcoxon test)	0.035	0.034	0.035	0.036	0.201	N/A

Note. (*a*) Subjects needed to score 10/12 correct trials in order to progress into the next test. Olivia, Oscar and Sam were tested for 10 sessions of stage 6a tasks and did not reach the criterion, therefore they were not tested for stage 6b. (*b*) The underlined subjects had breeding experience. A binomial test was performed for each individual subject and the subject needed to score at least 8/12 correct trials in the first session for the test to be significant (indicated by *). On the group level, a Wilcoxon test was performed for each stage to compare the group performance to the chance level (0.33).

Before this study, full object permanence had been previously demonstrated in birds belonging to the Corvidae family and the Psittaciforme order. In comparison to eight Goffin cockatoos (*Tanimbar corella*) tested using a similar method, Oriental pied hornbills were more consistent in the average number of trials required to meet the criterion. The cockatoos varied in the number of sessions required to pass a stage, ranging from 1.57 to 8.86 sessions [4]. Compared to the Eurasian jays (*Garrulus glandarius*) tested using the Uzgiris & Hunt scale [61], which is still based on the Piagetian framework, the jays seemed to outperform the hornbills, as all jays successfully completed all the tasks, with most tasks mastered within three sessions. However, two jays took longer due to developing fear of the experimental apparatus [26]. Hornbills on the other hand seem to be less neophobic than crows [62]. Many studies on object permanence in avian species have primarily focused on development (e.g. [9,63,64]). Future ontogenetic studies on hornbills could offer valuable insights into the development of this cognitive ability, and how this development compares to that of other avian species.

**Figure 2.**
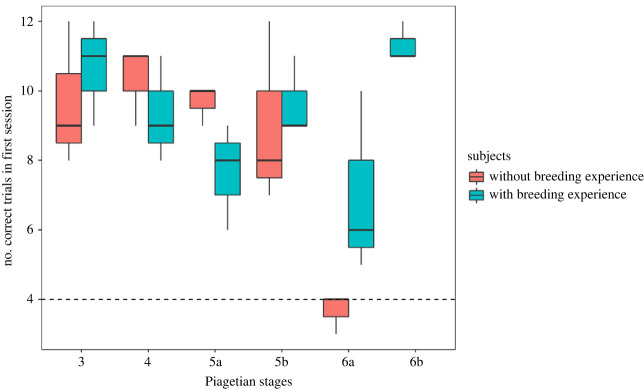
Number of correct choices in the first trials from stage 3 to 6a for subjects with and without breeding experience. Dashed line shows the number of correct choices expected by chance. *Group performance significantly above chance expectation.

One intriguing discovery in our study is that the three subjects who failed in the invisible displacement tasks lacked breeding experience, while those who passed had such experience. This raises the possibility that breeding experience might be a crucial factor in the development of object permanence in hornbills. Across all subjects, there were no significant performance differences between any two consecutive stages until stage 6a (Tukey HSD, *p* < 0.001). stage 6a appeared to pose more cognitive challenges for individuals without breeding experience (Tukey HSD, *p* < 0.001), but it did not significantly affect the performance of those with breeding experience (Tukey HSD, *p* = 0.23). Indeed, the performance of stage 6a was significantly lower than any of the previous stages for subjects without breeding experience, while it was only lower than stage 3 for subjects with breeding experience (figure 2; electronic supplementary material, tables S2 and table S3). This implies that breeding experience may be necessary for hornbills to understand invisible displacement but not visible displacement. When compared to visible displacement, understanding invisible displacement is more intricate, involving the integration of various cognitive skills, including memory, spatial reasoning and logical inference [65]. For hornbills, the experience of feeding a mate or chicks that cannot be seen or being fed by the male without visual contact may solidify their grasp of invisible displacement. It is also plausible that the development of object permanence is not solely a result of breeding experience. An alternative explanation for these results could be that they were influenced by the fact that the three specific subjects who failed the tasks were rescued birds that had fallen from the nest as fledglings. Oriental pied hornbills have an extended developmental period with parental care, staying in the nest for about two months before leaving when they are ready to fly [47,66]. Therefore, it is highly likely that these rescued subjects had not fully developed when they fell from the nest. If so, this suggests that the presence of fledglings in the nest with parental care may be critical for the full development of object permanence. However, it is essential to note that due to our small sample size of only six hornbills, the observed effect of breeding experience may have occurred purely by chance. Future more powered studies should be conducted to fully investigate the impact of both breeding behaviour and developmental periods on object permanence capability.

Our study has demonstrated that Oriental pied hornbills can exhibit full object permanence, contributing to expanding our knowledge in not only Piagetian object permanence in avian taxa but also cognition of Oriental pied hornbills. Though object permanence in hornbills is comparable to that of great apes, parrots and corvids, it has been pointed out that the same cognitive ability of different species can arise from very different underlying mechanisms [25]. The southern ground hornbill (*Bucorvus leadbeateri*), which is a sister clade to and fairly far removed from the Oriental pied hornbill [67], has been reported to demonstrate complex cognitive abilities, such as means-end understanding [68], reasoning by exclusion [69], reversal-learning [70] and social learning [71]. With their excellent performance in object permanence tasks, hornbills appear to be another highly cognitive avian species. However, further research is needed to assess whether hornbills possess cognitive abilities at a level similar to that of parrots and corvids.

## Data Availability

The datasets analysed during the current study and the code used are available from the OSF repository: https://osf.io/49kvt/ [72]. Supplementary material is available online [73].

## References

[1] Piaget J. 1954 The construction of reality in the child. New York, NY: Basic Books.

[2] Baillargeon R, Spelke ES, Wasserman S. 1985 Object permanence in five-month-old infants. Cognition **20**, 191-208. (10.1016/0010-0277(85)90008-3) 4064606

[3] Wellman HM, Cross D, Bartsch K, Harris PL. 1986 Infant search and object permanence: a meta-analysis of the A-not-B error. Monogr. Soc. Res. Child Dev. **51**, i-67. (10.2307/1166103) 3683418

[4] Auersperg AMI, Szabo B, von Bayern AMP, Bugnyar T. 2014 Object permanence in the Goffin cockatoo (*Cacatua goffini*). J. Comp. Psychol. **128**, 88-98. (10.1037/a0033272) 23875920

[5] Jaakkola K. 2014 Do animals understand invisible displacement? A critical review. J. Comp. Psychol. **128**, 225-239. (10.1037/a0035675) 24611640

[6] Trösch M, Flamand A, Chasles M, Nowak R, Calandreau L, Lansade L. 2020 Horses solve visible but not invisible displacement tasks in an object permanence paradigm. Front. Psychol. **11**, 562989. (10.3389/fpsyg.2020.562989) 33117229 PMC7552213

[7] de Blois ST, Novak MA, Bond M. 1998 Object permanence in orangutans (*Pongo pygmaeus*) and squirrel monkeys (*Saimiri sciureus*). J. Comp. Psychol. **112**, 137-152. (10.1037/0735-7036.112.2.137) 9642783

[8] Mendes N, Huber L. 2004 Object permanence in common marmosets (*Callithrix jacchus*). J. Comp. Psychol. **118**, 103-112. (10.1037/0735-7036.118.1.103) 15008678

[9] Pepperberg IM, Willner MR, Gravitz LB. 1997 Development of Piagetian object permanence in grey parrot (*Psittacus erithacus*). J. Comp. Psychol. **111**, 63-75. (10.1037/0735-7036.111.1.63) 9090138

[10] Redshaw M. 1978 Cognitive development in human and gorilla infants. J. Hum. Evol. **7**, 133-141. (10.1016/S0047-2484(78)80005-0)

[11] Triana E, Pasnak R. 1981 Object permanence in cats and dogs. Anim. Learn. Behav. **9**, 135-139. (10.3758/BF03212035)

[12] Doré FY, Dumas C. 1987 Psychology of animal cognition: Piagetian studies. Psychol. Bull. **102**, 219-233. (10.1037/0033-2909.102.2.219)

[13] Etienne AS. 1984 The meaning of object permanence at different zoological levels. Hum. Dev. **27**, 309-320. (10.1159/000272924)

[14] Parker ST. 1990 Origins of comparative developmental evolutionary studies of primate mental abilities. In ‘Language’ and intelligence in monkeys and apes: comparative developmental perspectives (eds KR Gibson, ST Parker), pp. 3-64. Cambridge, UK: Cambridge University Press.

[15] Smith MF, Watzek J, Brosnan SF. 2018 The Importance of a truly comparative methodology for comparative psychology. Int. J. Comp. Psychol. **31**. (10.46867/ijcp.2018.31.01.12)

[16] Anderson MR. 2012 Comprehension of object permanence and single transposition in gibbons. Behaviour **149**, 441-459. (10.1163/156853912X639769)

[17] Barth J, Call J. 2006 Tracking the displacement of objects: a series of tasks with great apes (*Pan troglodytes*, *Pan paniscus*, *Gorilla gorilla*, *and Pongo pygmaeus*) and young children (*Homo sapiens*). J. Exp. Psychol.: Anim. Behav. Process. **32**, 239-252. (10.1037/0097-7403.32.3.239) 16834492

[18] Call J. 2001 Object permanence in orangutans (*Pongo pygmaeus*), chimpanzees (*Pan troglodytes*), and children (*Homo sapiens*). J. Comp. Psychol. **115**, 159-171. (10.1037/0735-7036.115.2.159) 11459163

[19] de Blois ST, Novak MA. 1994 Object permanence in rhesus monkeys (*Macaca mulatta*). J. Comp. Psychol. **108**, 318-327. (10.1037/0735-7036.108.4.318) 1778064

[20] Deppe AM, Wright PC, Szelistowski WA. 2009 Object permanence in lemurs. Anim. Cogn. **12**, 381-388. (10.1007/s10071-008-0197-5) 18936991

[21] Fedor A, Skollár G, Szerencsy N, Ujhelyi M. 2008 Object permanence tests on gibbons (Hylobatidae). J. Comp. Psychol. **122**, 403-417. (10.1037/0735-7036.122.4.403) 19014264

[22] Mathieu M, Bouchard M-A, Granger L, Herscovitch J. 1976 Piagetian object-permanence in *Cebus capucinus*, *Lagothrica flavicauda* and *Pan troglodytes*. Anim. Behav. **24**, 585-588. (10.1016/S0003-3472(76)80071-1)

[23] Wood S, Moriarty KM, Gardner BT, Gardner RA. 1980 Object permanence in child and chimpanzee. Anim. Learn. Behav. **8**, 3-9. (10.3758/BF03209723)

[24] Pepperberg IM, Funk MS. 1990 Object permanence in four species of psittacine birds: an African grey parrot (*Psittacus erithacus*), an Illiger mini macaw (*Ara maracana*), a parakeet (*Melopsittacus undulatus*), and a cockatiel (*Nymphicus hollandicus*). Anim. Learn. Behav. **18**, 97-108. (10.3758/BF03205244)

[25] Pepperberg IM, Kozak FA. 1986 Object permanence in the African grey parrot (*Psittacus erithacus*). Anim. Learn. Behav. **14**, 322-330. (10.3758/BF03200074)

[26] Zucca P, Milos N, Vallortigara G. 2007 Piagetian object permanence and its development in Eurasian jays (*Garrulus glandarius*). Anim. Cogn. **10**, 243-258. (10.1007/s10071-006-0063-2) 17242935

[27] Dumas C, Wilkie DM. 1995 Object permanence in ring doves (*Streptopelia risoria*). J. Comp. Psychol. **109**, 142-150. (10.1037/0735-7036.109.2.142)

[28] Huffeldt NP. 2020 Performance of horned puffins (*Fratercula corniculata*) on an object permanence task. Behav. Processes **181**, 104274. (10.1016/j.beproc.2020.104274) 33069776

[29] Marhounová L, Frynta D, Fuchs R, Landová E. 2017 Object permanence in the food-storing coal tit (*Periparus ater*) and the non-storing great tit (*Parus major*): is the mental representation required? J. Comp. Psychol. **131**, 115-127. (10.1037/com0000061) 28263620

[30] Emery NJ, Clayton NS. 2004 The mentality of crows: convergent evolution of intelligence in corvids and apes. Science **306**, 1903-1907. (10.1126/science.1098410) 15591194

[31] Güntürkün O, Bugnyar T. 2016 Cognition without cortex. Trends Cogn. Sci. **20**, 291-303. (10.1016/j.tics.2016.02.001) 26944218

[32] Lambert ML, Jacobs I, Osvath M, von Bayern AMP. 2019 Birds of a feather? Parrot and corvid cognition compared. Behaviour **156**, 505-594. (10.1163/1568539X-00003527)

[33] Auersperg AMI, Laumer IB, Bugnyar T. 2013 Goffin cockatoos wait for qualitative and quantitative gains but prefer ‘better’ to ‘more’. Biol. Lett. **9**, 20121092. (10.1098/rsbl.2012.1092) 23485873 PMC3645019

[34] Dufour V, Pelé M, Sterck EHM, Thierry B. 2007 Chimpanzee (*Pan troglodytes*) anticipation of food return: coping with waiting time in an exchange task. J. Comp. Psychol. **121**, 145-155. (10.1037/0735-7036.121.2.145) 17516793

[35] Hillemann F, Bugnyar T, Kotrschal K, Wascher CAF. 2014 Waiting for better, not for more: corvids respond to quality in two delay maintenance tasks. Anim. Behav. **90**, 1-10. (10.1016/j.anbehav.2014.01.007) 25892738 PMC4398876

[36] Hill A, Collier-Baker E, Suddendorf T. 2011 Inferential reasoning by exclusion in great apes, lesser apes, and spider monkeys. J. Comp. Psychol. **125**, 91-103. (10.1037/a0020867) 21341913

[37] Mikolasch S, Kotrschal K, Schloegl C. 2011 African grey parrots (*Psittacus erithacus*) use inference by exclusion to find hidden food. Biol. Lett. **7**, 875-877. (10.1098/rsbl.2011.0500) 21697165 PMC3210682

[38] Schloegl C. 2011 What you see is what you get—reloaded: can jackdaws (*Corvus monedula*) find hidden food through exclusion? J. Comp. Psychol. **125**, 162-174. (10.1037/a0023045) 21604851

[39] Clayton NS, Dickinson A. 1998 Episodic-like memory during cache recovery by scrub jays. Nature **395**, 272-274. (10.1038/26216) 9751053

[40] Auersperg AMI, Szabo B, von Bayern AMP, Kacelnik A. 2012 Spontaneous innovation in tool manufacture and use in a Goffin's cockatoo. Curr. Biol. **22**, R903-R904. (10.1016/j.cub.2012.09.002) 23137681

[41] Sousa C, Biro D, Matsuzawa T. 2009 Leaf-tool use for drinking water by wild chimpanzees (*Pan troglodytes*): acquisition patterns and handedness. Anim. Cogn. **12**, 115-125. (10.1007/s10071-009-0278-0) 19697068

[42] Weir AAS, Chappell J, Kacelnik A. 2002 Shaping of hooks in New Caledonian crows. Science **297**, 981. (10.1126/science.1073433) 12169726

[43] Emery NJ. 2006 Cognitive ornithology: the evolution of avian intelligence. Phil. Trans. R. Soc. B **361**, 23-43. (10.1098/rstb.2005.1736) 16553307 PMC1626540

[44] Iwaniuk AN, Dean KM, Nelson JE. 2004 Interspecific allometry of the brain and brain regions in parrots (Psittaciformes): comparisons with other birds and primates. Brain Behav. Evol. **65**, 40-59. (10.1159/000081110) 15467290

[45] Lefebvre L, Aurora G, Sherry D, Sarah T, Lajos R, Peter K. 1998 Feeding innovations and forebrain size in Australasian birds. Behaviour **135**, 1077-1097. (10.1163/156853998792913492)

[46] Emery NJ, Clayton NS. 2004 Comparing the complex cognition of birds and primates. In Comparative vertebrate cognition: are primates superior to non-primates? (eds LJ Rogers, G Kaplan), pp. 3-55. Boston, MA: Springer.

[47] Kemp A. 2007 Hornbills. In The new encyclopedia of birds (ed. C Perrins). Oxford, UK: Oxford University Press.

[48] Bond AB, Kamil AC, Balda RP. 2003 Social complexity and transitive inference in corvids. Anim. Behav. **65**, 479-487. (10.1006/anbe.2003.2101)

[49] Diamond J, Bond A. 2003 A comparative analysis of social play in birds. Behaviour **140**, 1091-1115. (10.1163/156853903322589650)

[50] Kasambe R, Charde P, Yosef R. 2011 Aerial jousting and bill grappling in Indian grey hornbill (*Ocyceros birostris*). Acta Ethol. **14**, 13-15. (10.1007/s10211-010-0085-2)

[51] Chong JL, Yeap CA, Yong JWH, Kumaran JV, Nelson BR, Loh IH. 2022 Ethological evidence of adaptive predation of Oriental pied hornbill (*Anthracoceros albirostris*) on farmed swiftlet (*Aerodramus* spp.) in Kalabakan, Sabah Malaysia. Acta Ecologica Sinica **42**, 255-258. (10.1016/j.chnaes.2021.08.007)

[52] Yeap C-A, Perumal B. 2017 Distribution of hornbills and important hornbill landscapes: setting site conservation priorities for Peninsular Malaysia. In 7th Int. Hornbill Conf. 2017 “Hornbills: Fly Free Fly High”, 16–18 May 2017, Sarawak, Malaysia.

[53] Ismail A, Jamil N, Rahman F. 2015 The use of abandoned clay jars for nesting by Oriental-pied hornbill in Sungai Panjang, Sabak Bernam. Malayan Nature Journal **67**, 42-49.

[54] Chaiyarat R, Kongprom U, Manathamkamon D, Wanpradab S, Sangarang S. 2012 Captive breeding and reintroduction of the oriental pied hornbill (*Anthracoceros albirostris*) in Khao Kheow Open Zoo, Thailand. Zoo Biol. **31**, 683-693. (10.1002/zoo.20432) 22105510

[55] Loong S, Sin YCK, Johns P, Plowden T, Yong DL, Lee J, Jain A. 2021 Nest predation by Oriental pied hornbills *Anthracoceros albirostris* in urban Singapore. BirdingAsia **35**, 86-91.

[56] Shukla U, Prasad S, Joshi M, Sridhara S, Westcott DA. 2015 Nest site characterization of sympatric hornbills in a tropical dry forest. Curr. Sci. **108**, 1725-1730.

[57] Kalina J. 1989 Nest intruders, nest defence and foraging behaviour in the black-and-white casqued hornbill *Bycanistes subcylindricus*. Ibis **131**, 567-571. (10.1111/j.1474-919X.1989.tb04791.x)

[58] RStudio Team. 2020 RStudio: integrated development environment for R. Boston, MA: RStudio, PBC.

[59] Bates D, Mächler M, Bolker B, Walker S. 2015 Fitting linear mixed-effects models using lme4. J. Stat. Softw. **67**, 1-48. (10.18637/jss.v067.i01)

[60] Lenth RV. 2022 emmeans: Estimated Marginal Means, aka Least-Squares Means. R package version 1.8.1-1. See https://CRAN.R-project.org/package=emmeans

[61] Uzgiris IC, Hunt JM. 1975. Assessment in infancy: ordinal scales of psychological development. Champaign, IL: University of Illinois Press.

[62] Garcia-Pelegrin E, Clark F, Miller R. 2022 Increasing animal cognition research in zoos. Zoo Biol. **41**, 281-291. (10.1002/zoo.21674) 35037289

[63] Hoffmann A, Rüttler V, Nieder A. 2011 Ontogeny of object permanence and object tracking in the carrion crow, *Corvus corone*. Anim. Behav. **82**, 359-367. (10.1016/j.anbehav.2011.05.012)

[64] Ujfalussy DJ, Miklósi Á, Bugnyar T. 2013 Ontogeny of object permanence in a non-storing corvid species, the jackdaw (*Corvus monedula*). Anim. Cogn. **16**, 405-416. (10.1007/s10071-012-0581-z) 23161215 PMC4417713

[65] Cacchione T, Rakoczy H. 2017 Comparative metaphysics: thinking about objects in space and time. In APA handbook of comparative psychology: perception, learning, and cognition, Vol. 2, pp. 579-599. Washington, DC: American Psychological Association.

[66] Ng S-C, Lai HM, Cremades M, Lim MTS, Tali SB. 2011 Breeding observations on the Oriental pied hornbill in nest cavities and in artificial nests in Singapore, with emphasis on infanticide-cannibalism. Baffles Bull. Zool. **24**, 15-22.

[67] Viseshakul N, Charoennitikul W, Kitamura S, Kemp A, Thong-Aree S, Surapunpitak Y, Poonswad P, Ponglikitmongkol M. 2011 A phylogeny of frugivorous hornbills linked to the evolution of Indian plants within Asian rainforests. J. Evol. Biol. **24**, 1533-1545. (10.1111/j.1420-9101.2011.02285.x) 21545425

[68] Danel S, von Bayern AMP, Osiurak F. 2019 Ground-hornbills (*Bucorvus*) show means-end understanding in a horizontal two-string discrimination task. J. Ethol. **37**, 117-122. (10.1007/s10164-018-0565-9)

[69] Danel S, Rebout N, Kemp LV. 2022 Through the eyes of a hunter: assessing perception and exclusion performance in ground-hornbills. Anim. Cogn. **25**, 1665-1670. (10.1007/s10071-022-01619-3) 35394265

[70] Danel S, Rebout N, Kemp L. 2023 Assessing sex differences in behavioural flexibility in an endangered bird species: the Southern ground-hornbill (*Bucorvus leadbeateri*). Anim. Cogn. **26**, 599-609. (10.1007/s10071-022-01705-6) 36251104

[71] Danel S, Rebout N, Kemp L. 2023 Social diffusion of new foraging techniques in the Southern ground-hornbill (*Bucorvus leadbeateri*). Learn. Behav. **51**, 153-165. (10.3758/s13420-022-00518-4) 35230667

[72] Yao R, Garcia-Pelegrin E. 2024 Oriental pied hornbills (*Anthracoceros albirostris*) solve invisible displacement tasks in a test of Piagetian object permanence. OSF repository. (https://osf.io/49kvt/) 10.1098/rsbl.2023.0547 PMC10827421 38290552

[73] Yao R, Garcia-Pelegrin E. 2024 Oriental pied hornbills (*Anthracoceros albirostris*) solve invisible displacement tasks in a test of Piagetian object permanence. *Figshare*. (10.6084/m9.figshare.c.7041571) PMC10827421 38290552

